# Four new species of the millipede genus *Eutrichodesmus* Silvestri, 1910 from Laos, including two with reduced ozopores (Diplopoda, Polydesmida, Haplodesmidae)

**DOI:** 10.3897/zookeys.660.11780

**Published:** 2017-03-08

**Authors:** Weixin Liu, Sergei Golovatch, Thomas Wesener

**Affiliations:** 1 Zoological Research Museum A. Koenig, Leibniz Institute for Animal Biodiversity, Adenauerallee 160, Bonn 53113, Germany; 2 Department of Entomology, College of Agriculture, South China Agricultural University, 483 Wushanlu, Guangzhou 510642, China; 3 Institute for Problems of Ecology and Evolution, Russian Academy of Sciences, Leninsky pr. 33, Moscow 119071, Russia

**Keywords:** Millipede, reduced ozopores, biodiversity, taxonomy, cave adaptation, Laos

## Abstract

Laos has large areas of primary forest with a largely unexplored fauna. This is evidenced by millipedes, class Diplopoda, with fewer than 60 species being recorded from the country. In the widespread Southeast Asian “Star Millipede” genus *Eutrichodesmus* Silvestri, 1910 (family Haplodesmidae), only two of 49 recorded species have been found in Laos. Four new species of Star Millipedes are here described from caves in Laos: *Eutrichodesmus steineri* Liu & Wesener, **sp. n.**, *Eutrichodesmus deporatus* Liu & Wesener, **sp. n.**, *Eutrichodesmus paraster* Liu & Wesener, **sp. n.** and *Eutrichodesmus parvus* Liu & Wesener, **sp. n.**. A fifth species, for which only a female is available, remains unnamed. The defensive glands (ozopores) are found to be strongly or entirely suppressed in two of the new species, *Eutrichodesmus deporatus* Liu & Wesener, **sp. n.** and *Eutrichodesmus paraster* Liu & Wesener, **sp. n.**, both troglobionts, which is new to the family. All of the Star Millipedes were collected during Northern Lao-European Cave Project faunal surveys conducted by the Senckenberg Museum, Frankfurt. A key to the six species of *Eutrichodesmus* currently known to occur in Laos is provided.

## Introduction

The documenting of biodiversity and the subsequent taxonomic descriptions of undescribed species have been highlighted as one of the most urgent research programmes of our planet, as indicated by the declaration of the “United Nations Decade on Biodiversity”, as well as the signing of the UN “Convention on Biological Diversity” by numerous countries (e.g., Wheeler 2008; Padial et al. 2010; Popescu 2015). Laos, a landlocked, largely montane, tropical country in Southeast Asia, is one of the highly biodiverse Great Mekong countries, which together have yielded more than 2200 new species since 1997 (WWF 2016). Furthermore, in contrast to its neighbours China, Vietnam and Thailand, the forests in Laos still remain relatively intact (STEA 2000). However, this might change in the near future as illegal logging and timber smuggling to Vietnam are being conducted on a large scale (EIA 2011; Smirnov 2015; Gan et al. 2016). The very large amount of still undescribed biodiversity in Laos is especially evident in arthropods, including the large, ecologically important, mostly sylvicolous and mesophilous millipedes, class Diplopoda. Most diplopods are detritivores whose primary habitat is forest litter and topsoil, but many species live in caves, dead wood, suspended soil or even tree canopies (e.g., Golovatch and Kime 2009). Diplopoda are an ancient, diverse and widespread group, with fossils dating back to the Silurian (Edgecombe 2015) and with about 12,000 described species in >3,000 recognized genera, >150 families and 16 orders (Minelli 2015). Since the bulk of global millipede diversity is confined to tropical forest, which is a rapidly shrinking biome, and because diplopods are poor dispersers that are largely confined to forests and woodlands, and are prone to strongly localized endemism (e.g. Wesener 2009; Car and Harvey 2014; Enghoff 2015), the problem of documenting millipede faunas is increasingly acute (Golovatch and Kime 2009). These localized occurrences make millipede species important subjects for biogeographic studies (Stoev and Enghoff 2003; Wesener et al. 2010; Wesener et al. 2011), but also put them at risk of local extinction from human activities such as forest destruction or large-scale mining operations (Wesener and Wägele 2007; Iniesta et al. 2012). This holds especially true for Laos where the pace of forest destruction is alarmingly high (Gan et al. 2016).

Fortunately, fresh collections of Laotian millipedes have encouraged recent taxonomic studies, and since the latest checklist for the country which listed 34 species (Likhitrakarn et al. 2014a), another 23 have been added (Likhitrakarn et al. 2014a, 2014b, 2014c, 2015a, 2015b, 2016a, 2016b; Golovatch 2016a, 2016b; Golovatch et al. 2016a, 2016b). Still, the achievement is modest, as the faunas of the adjacent Vietnam, Thailand and southern China comprise from >100 to a few hundred millipede species each (e.g., Enghoff et al. 2004; Enghoff 2005; Golovatch 2015). In addition, only seven of the 16 orders of Diplopoda have been recorded so far in Laos, the most species-rich being the Polydesmida.

**Figure 1.**
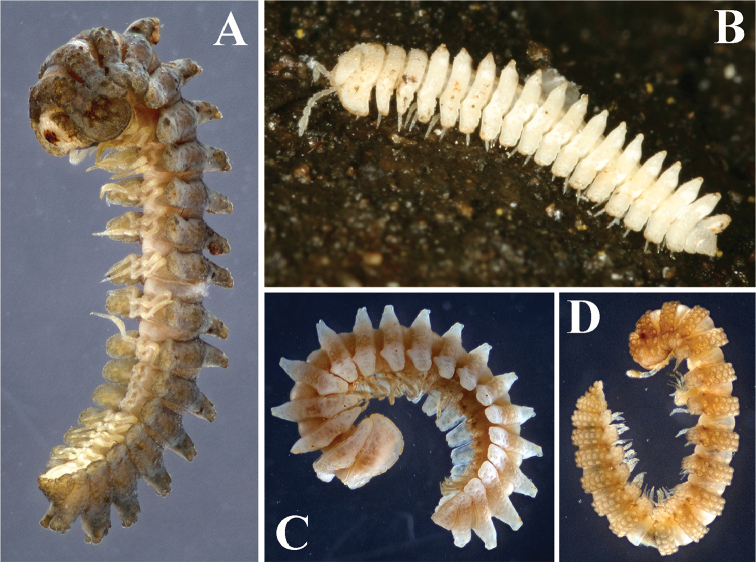
Habitus photographs. **A** *Eutrichodesmus steineri* sp. n., male paratype (SMF) from Cave Tham Dout, ventrolateral view **B** *Eutrichodesmus deporatus* sp. n., a male ecological photo from Cave Tham Pathok, sublateral view **C** *Eutrichodesmus paraster* sp. n., male holotype (SMF) from Cave Tham Long Puang, lateral view **D** *Eutrichodesmus parvus* sp. n., male paratype (ZFMK) from Cave Tham Nam Long, lateral view.

Within the Polydesmida, the tiny species of “Star Millipedes”, genus *Eutrichodesmus* Silvestri, 1910 (family Haplodesmidae), with their often conspicuous dorsal projections (e.g., Fig. 1), together with the larger “Dragon Millipedes”, genus *Desmoxytes* Chamberlin, 1923 (family Paradoxosomatidae, see Liu et al. 2014, 2016), are among the most remarkable diplopods in Southeast Asia. *Eutrichodesmus* is one of the most speciose genera of SE Asian millipedes, presently containing 49 described species (Golovatch et al. 2015, 2016a). The genus is distributed from southern Japan in the north, through southern China and Indochina, to Vanuatu, Melanesia in the south. Most species are strongly localized country endemics, this being especially true of the rather numerous cavernicoles. Laos is situated more or less north-centrally within the distribution range of the genus, but only two Laotian species have been named so far: *Eutrichodesmus multilobatus* Golovatch, Geoffroy, Mauriès & VandenSpiegel, 2009, and *Eutrichodesmus nadan* Golovatch, Geoffroy, Mauriès & VandenSpiegel, 2016. Both are highly localized endemics found in caves and are presumed troglobites (Golovatch et al. 2009a, 2016a).

Below we describe four new species and provide a key to all six members of *Eutrichodesmus* currently known to occur in Laos. In addition, for the first time in the family we report strongly or completely suppressed ozopores, which is unusual because all previously described *Eutrichodesmus* seem to show normal pore formulae: 5, 7, 9, 10, 12, 13, 15–19.

## Material and methods

Specimens were collected for the Northern Lao-European Cave Project, and kept in 70% ethanol. The holotypes and a number of paratypes are deposited in the zoological collection of the Senckenberg Research Institute and Natural History Museum (SMF), with some material also to be housed in the Zoological Research Museum A. Koenig (ZFMK).

Observation and dissections were performed using an Olympus SZ51 stereo microscope. The line drawings were prepared with the help of an Olympus BX51 microscope and an attached camera for the scope. SEM micrographs were taken using a ZEISS Sigma 300VP scanning electron microscope (based at the ZFMK). Dry SEM material was coated with gold, removed after study from stubs and returned to alcohol. The photographs were taken with Canon EOS 7D cameras and further processed using Adobe Photoshop CS6 software.

The terminology used here follows that of Golovatch et al. (2009a, 2009b).

### Abbreviations used

**SMF** Senckenberg Research Institute and Natural History Museum, Frankfurt am Main, Germany

**SEM** Scanning electron microscopy

**ZFMK** Zoological Research Museum Alexander Koenig, Bonn, Germany

## Taxonomy

### A key to species of *Eutrichodesmus* in Laos

**Table:** 

1	Habitus in lateral view resembling a star: metaterga 5–19 each with a very high, mid-dorsal projection (Fig. 1A–C)	**2**
–	Habitus non-asteriform: metaterga 5–19 devoid of mid-dorsal projections (Fig. 1D)	**4**
2	Metatergum 4 with a high mid-dorsal projection (Figs 1C, 8E). Gonopod with a large, lateral, denticulate, distofemoral process (**dp**); acropodite with a very small mesal tooth (**t**) subapically (Fig. 10)	***Eutrichodesmus paraster* sp. n.**
–	Metatergum 4 devoid of a high mid-dorsal projection. Gonopod with a prominent, digitiform, distofemoral process (**dp**); acropodite with a micropapillate process (**pp**) near midway or at base (Figs 4, 7)	**3**
3	Body larger, about 9.5–10.0 mm long, grey-brown in colour. Ozopores distinct (Figs 2E, 3H), pore formula normal. Seminal groove on gonopod terminating at a mesal lobule (**lo**) subapically (Figs 3N, 4)	***Eutrichodesmus steineri* sp. n.**
–	Body smaller, about 7.5–8.0 mm long, uniformly pallid. Ozopores strongly reduced, only visible on paraterga 17 (Fig. 6C, G). Gonopod acropodite subapically with a slightly bifid, dorsolateral tooth (**t1**); seminal groove terminating at a small triangular tooth (**t2**) subapically (Fig. 7)	***Eutrichodesmus deporatus* sp. n.**
4	Paraterga 5-lobulated laterally. Gonopod very simple, acropodite devoid of any tooth or lobe	***Eutrichodesmus multilobatus* Golovatch, Geoffroy, Mauriès & VandenSpiegel, 2009**
–	Paraterga 2- or 3-lobulated laterally. Gonopod relatively complex, acropodite with a tooth or lobe subapically	**5**
5	Body conglobation complete, with laterally bilobate paraterga. Tip of gonopod acropodite subunciform, with a small, mesal, subapical lobule; seminal groove terminating in an evident accessory seminal chamber, with a distinct hairpad proximal to it	***Eutrichodesmus nadan* Golovatch, Geoffroy, Mauriès & VandenSpiegel, 2016**
–	Body conglobation incomplete, with laterally mostly trilobate paraterga. Gonopod acropodite with a small tooth (**t**) dorsally and an evident, digitiform lobe (**lo**) ventrally; seminal groove terminating without hairpad (Fig. 13)	***Eutrichodesmus parvus* sp. n.**

#### Eutrichodesmus steineri

Taxon classificationAnimaliaPolydesmidaHaplodesmidae

Liu & Wesener sp. n.

http://zoobank.org/C94274F9-16D8-41E0-8B38-C8DC6C7A6678

Figs 1A , 2 , 3 , 4

##### Material examined.

Holotype male (SMF), Laos, Luang Prabang Province, Phou Khoun District, Cave Tham Deu (E 48-013-005), N19°26'4.3", E102°29'16.6", 6.I.2007, coll. L. Price (205/07-).

##### Paratypes.

1 male (ZFMK MYR6130), 2 juveniles (ZFMK MYR6126), same data as holotype; 1 male, 5 females, 7 juveniles (SMF), same locality (E 48-013-005), 5.I.2007, coll. H. Steiner (210/07-); 1 female (ZFMK MYR6133), same data as above; 1 male, 1 female, 1 juvenile (SMF), same district, Cave Tham Dout (E 48-013-004), 5.I.2007, coll. L. Price (139/07-).

##### Etymology.

Honours Mr. H. Steiner, one of the collectors; noun.

##### Diagnosis.

Differs from other species of the genus in showing laterally 3-lobulated paraterga and the extremely high mid-dorsal projections on metaterga 5–19, the latter character very similar to that observed in *Eutrichodesmus macclurei* Hoffman, 1977, from western Malaysia (Hoffman 1977). However, *Eutrichodesmus steineri* sp. n. is distinct from *Eutrichodesmus macclurei* in the gonopod, which has a long, digitiform, distofemoral process, *vs.* a short spiniform process in the counterpart. See also Key above.

##### Description.

Length of adults of both sexes ca. 9.5–10.0 mm, width 0.8–1.0 mm and 2.0–2.2 mm on midbody pro- and metazona, respectively.

Coloration uniformly grey-brown with pallid antennae (Fig. 1A).

Adults with 20 segments (Fig. 1A), body subcylindrical, conglobation complete.

**Figure 2.**
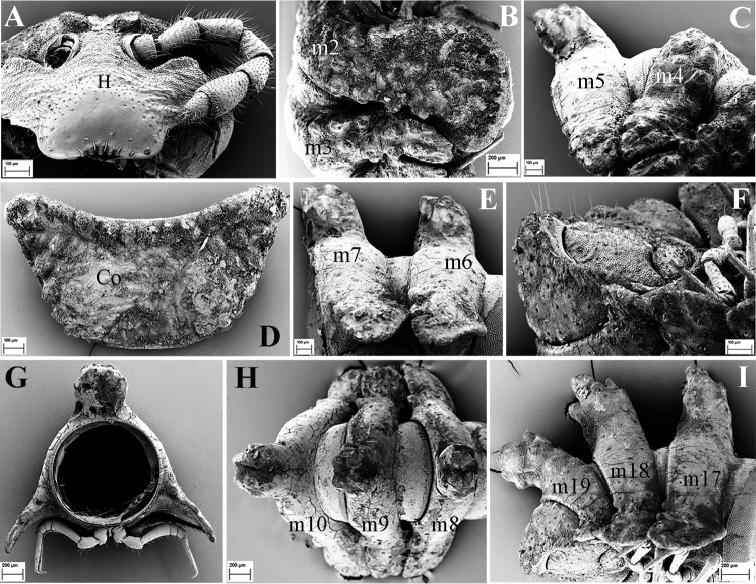
*Eutrichodesmus steineri* sp. n., SEM, male paratype from Cave Tham Deu. **A** head (H) and left antenna, frontal view **B** segments 2 and 3, lateral view **C.** segments 4 and 5, lateral view (m2–m5 = metaterga 2–5) **D** collum (Co), dorsal view **E** segments 6 and 7, lateral view (m6–m7 = metaterga 6, 7) **F** telson, subventral view **G** cross-section of segment 11, caudal view **H** segments 8–10, dorsal view **I** segments 17–19 and telson, lateral view (m8–m10, m17–m19 = metaterga 8–10, 17–19).

Head slightly transverse, frons densely pilose, microgranular except for clypeus, with a paramedian pair of rounded, paramedian, microvillose knobs above antennal sockets (Fig. 2A). Epicranial suture conspicuous.

**Figure 3.**
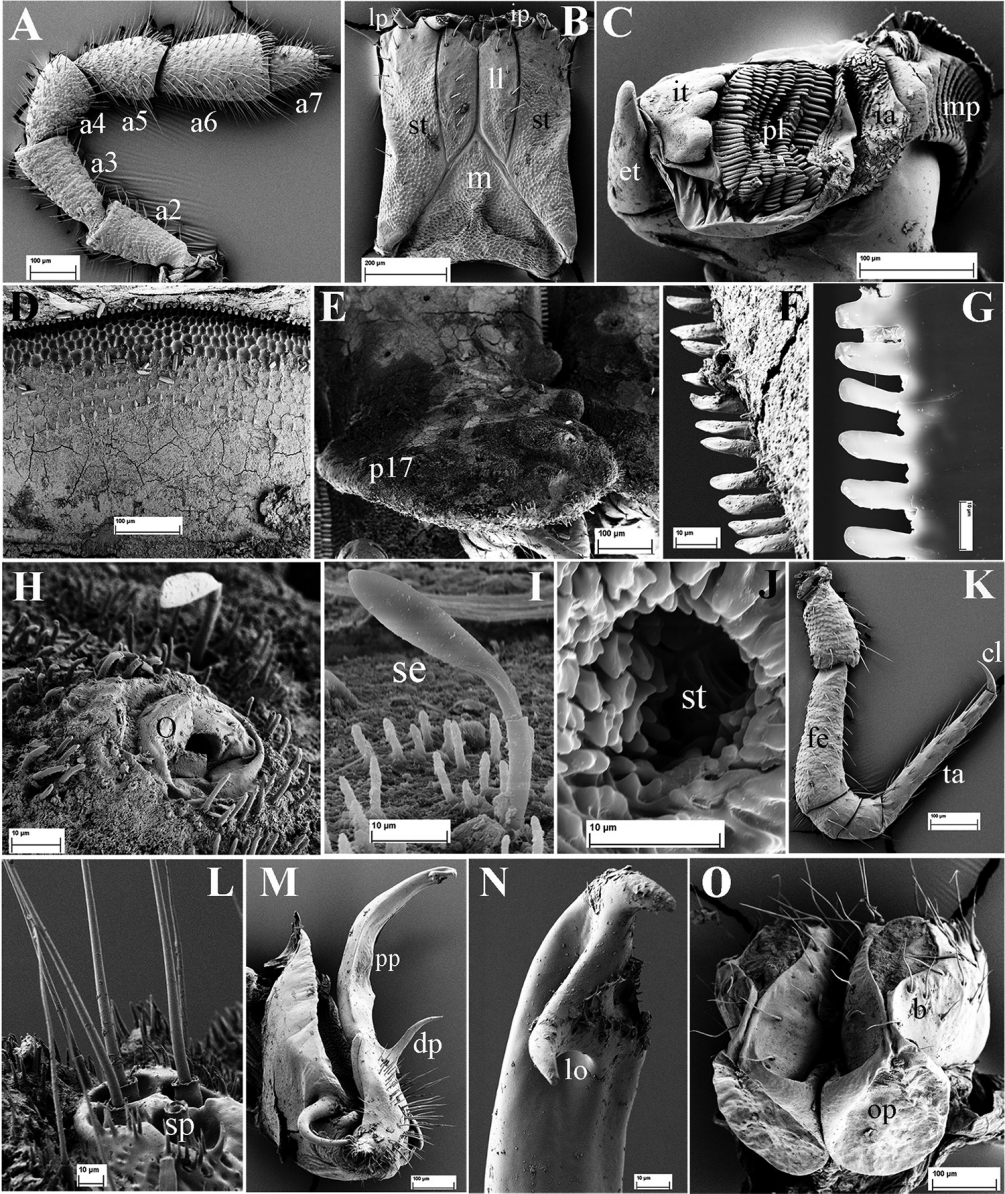
*Eutrichodesmus steineri* sp. n., SEM, male paratype from Cave Tham Deu. **A** right antenna, lateral view (a2–a7 = antennomeres 2–7) **B** gnathochilarium, ventral view (lp = lateral palpus; ip = inner palpus; st = stipites; ll = lamellae linguales; m = mentum) **C** right mandible, general view (et = external tooth; it = internal tooth; pl = pectinate lamellae; ia = intermediate area; mp = molar plate) **D** prozonum 8, dorsal view **E** paratergum 17 (p17), lateral view **F** limbus of metatergum 5, lateral view **G** endotergum 7 **H** ozopore (o) and a seta of paratergum 17, general view **I** a seta (se) **J** stigmata (st) on segment 6, ventral view **K** midbody leg, frontal view (fe = femur; ta = tarsus; cl = claw) **L** spinnerets (sp), subventral view **M** left gonopod, mesal view (dp = distofemoral process; pp = papillate process) **N** tip of left gonopod, mesal view (lo = lobule) **O** female paratype, vulvae, general view (op = operculum; b = bursa).

Antennae densely pilose, short, but slender, only slightly clavate (Figs 2A, 3A). In length, antennomere 6 > 3 > 2 > 4 = 5 > 7 > 1. Antennomeres 5 and 6 each with an evident group of minute bacilliform sensilla dorso-apically; disc with four sensory cones apically (Figs 2A, 3A).

Labrum usually with three, rarely five teeth, lateral ones smaller (Fig. 2A).

Gnathochilarium (Fig. 3B) with a long bacilliform sensillum apically on each lamella lingualis (**ll**); mentum (**m**) triangular.

Mandible with a movable external tooth (**et**), an internal tooth (**it**) with four cusps; six pectinate lamellae (**pl**) consisting of long, smooth teeth; intermediate area (**ia**) covered with small cuticular scales; a large, stairs-like molar plate (**mp**) close to anterior fringe with pin-like structures (Fig. 3C).

Collum subtrapeziform (Fig. 2D), slightly broader than head, not covering the latter from above; dorsal surface with six transverse rows of round microvillose tubercles, flattened medially (Fig. 2D); each tubercle crowned by a 2-segmented seta, these setae being mostly abraded. Frontal margin slightly elevated (Fig. 2D).

Prozona very finely alveolate; stricture between pro- and metazona broad, shallow and smooth (Fig. 3D). Limbus regularly crenulate (Fig. 3F–G). Endotergum smooth (Fig. 3G).

Metaterga 2–4 each with three transverse mixostictic rows of similar small tubercles extending onto paraterga (Fig. 2B–C), 7(8) + 7(8) per row. Three transverse rows of very small, shallow, microvillose tuberculations on metaterga 5–19 (Fig. 2C, E, H–I), while metaterga 5–19 with a very high, large, mid-dorsal projection bifid on each side (Figs 1A, 2C, E, G–I). Projections 5–17 directed upright, then inclined slightly caudad on metaterga 18 and 19 (Fig. 2I). Metatergal setae 2-segmented, often abraded (Fig. 3H–I).

Paraterga with evident shoulders anteriorly, strongly declivous, broad and usually trilobate laterally (Figs 2H–I, 3E), evidently extending down below level of venter (Fig. 2G); caudolaterally at base with two distinct lobulations (Figs 2H–I, 3E). Paraterga 2 strongly enlarged, a lateral lobulation indistinct, but two caudolateral lobulations evident (Fig. 2B); paraterga 3 and 4 slightly shorter than others, bilobate laterally (Fig. 2B).

Pore formula normal (5, 7, 9, 10, 12, 13, 15–19), ozopores distinct, each located near top of caudolateral lobulation (Figs 2E, 3E, H).

Pre-anal ring short, with four transverse rows of very small and flat tuberculations (Fig. 2F, I). Epiproct apically with four spinnerets (Fig. 3L). Paraprocts and hypoproct densely microvillose; paraprocts with two pairs of long setae, hypoproct subtrapeziform, with two long setae (Fig. 2F).

Pleurosternal keels absent. Sterna very narrow (Fig. 2G), but much broader only between male coxae 6–7 and 9. Stigmata clearly visible (Fig. 3J). Gonopod aperture suboval.

Legs long and slender, nearly reaching tips of paraterga (Fig. 2G); tarsus longer than femur; claw simple, curved ventrad (Fig. 3K).

**Figure 4.**
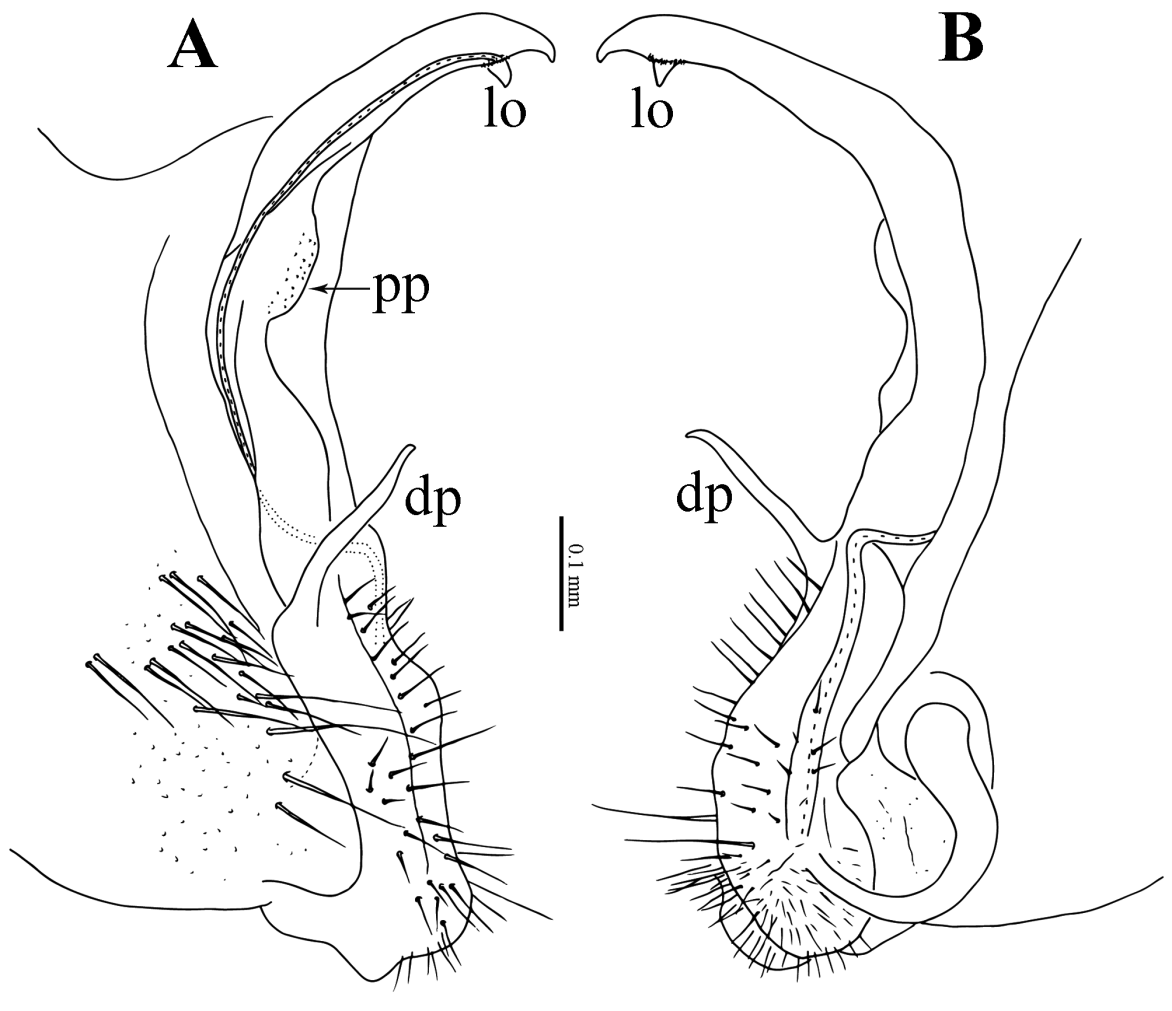
*Eutrichodesmus steineri* sp. n., male paratype from Cave Tham Deu. **A–B** right gonopod, sublateral and mesal views, respectively. Abbreviations: dp = distofemoral process; pp = papillate process; lo = lobule.

Gonopods (Figs 3M–N, 4) simple. Coxae large, abundantly micropapillate and setose ventrolaterally. Telopodite slightly longer than coxite, slender throughout, subfalcate, distinctly curved ventrad, setose in its basal part, with a prominent, digitiform, lateral, distofemoral process (**dp**) at about basal one-third. Acropodite with a micropapillate process (**pp**) at midway; seminal groove long, terminating in a hairpad at a small, triangular, mesal lobule (**lo**) subapically.

Vulvae lying inside a membranous sac, each vulva consisting of a large horseshoe-shaped operculum (**op**) and a bursa (**b**) with several long setae (Fig. 3O).

#### Eutrichodesmus deporatus

Taxon classificationAnimaliaPolydesmidaHaplodesmidae

Liu & Wesener sp. n.

http://zoobank.org/F01FD071-6226-4A7F-A3AB-45FCFF7FFA1E

Figs 1B , 5 , 6 , 7

##### Material examined.

Holotype male (SMF), Laos, Luang Prabang Prov., NE Luang Prabang, Nam Ou, Nong Khiao, Cave Tham Pathok, hand collected, N20°33.082', E102°37.925', 373 m, 11.III.2006, coll. P. Jäger & J. Altmann.

##### Paratypes.

1 male, 1 female (ZFMK MYR6128 & 6129), same data as holotype; 2 females, 3 juveniles (SMF), same data as holotype; 1 male (SMF), same locality, 29.II.2008, coll. P. Jäger.

##### Etymology.

To emphasize the ozopores in this species being mostly reduced; adjective.

##### Diagnosis.

Differs from all other species of the genus in the ozopores retained only on body segment 17, coupled with the gonopod acropodite showing a slightly bifid dorsolateral tooth subapically. See also Key above.

##### Description.

Length of adults of both sexes ca. 7.5–8.0 mm, width 0.6–0.8 mm and 1.6–1.8 mm on midbody pro- and metazona, respectively.

Coloration uniformly pallid (Fig. 1B).

Adults with 20 segments (Fig. 1B), body conglobation complete.

**Figure 5.**
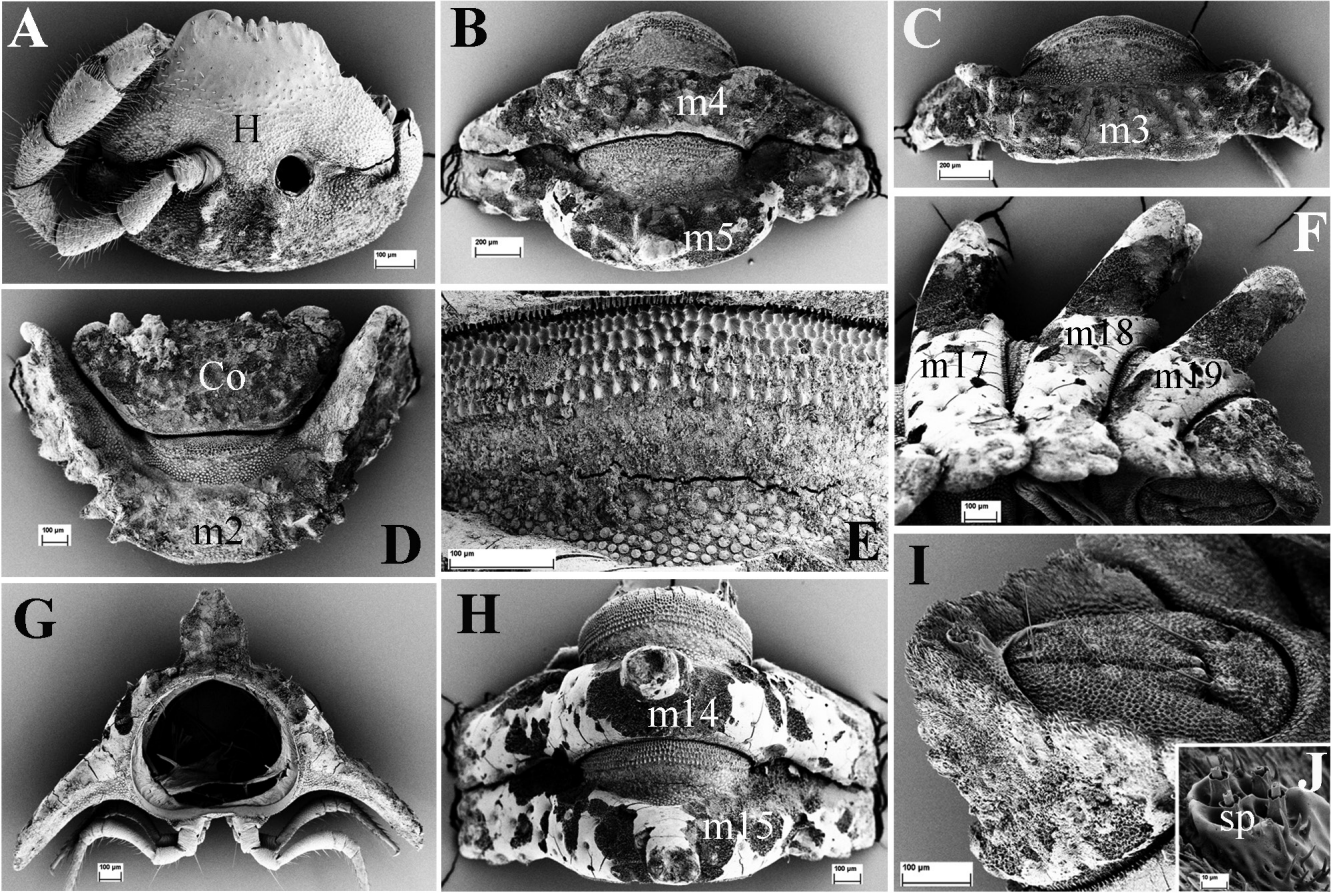
*Eutrichodesmus deporatus* sp. n., SEM, male paratype from Cave Tham Pathok. **A** head (H) and left antenna, frontal view **B** segments 4 and 5, dorsal view **C** segment 3, dorsal view (m3–m5 = metaterga 3–5 **D** collum (Co) and segment 2 (m2 = metatergum 2), dorsal view **E** prozonum 15, dorsal view **F** segments 17–19 and telson, lateral view (m17–m19 = metaterga 17–19) **G** cross-section of segment 6, caudal view **H** segments 14 and 15, dorsal view (m14–m15 = metaterga 14–15) **I** telson, subventral view **J** spinnerets (sp), subventral view.

Antennae short, but slender (Figs 1B, 5A); in length, antennomere 6 > 3 = 2 > 4 = 5 > 7 > 1.

Labrum with three teeth (Fig. 5A).

**Figure 6.**
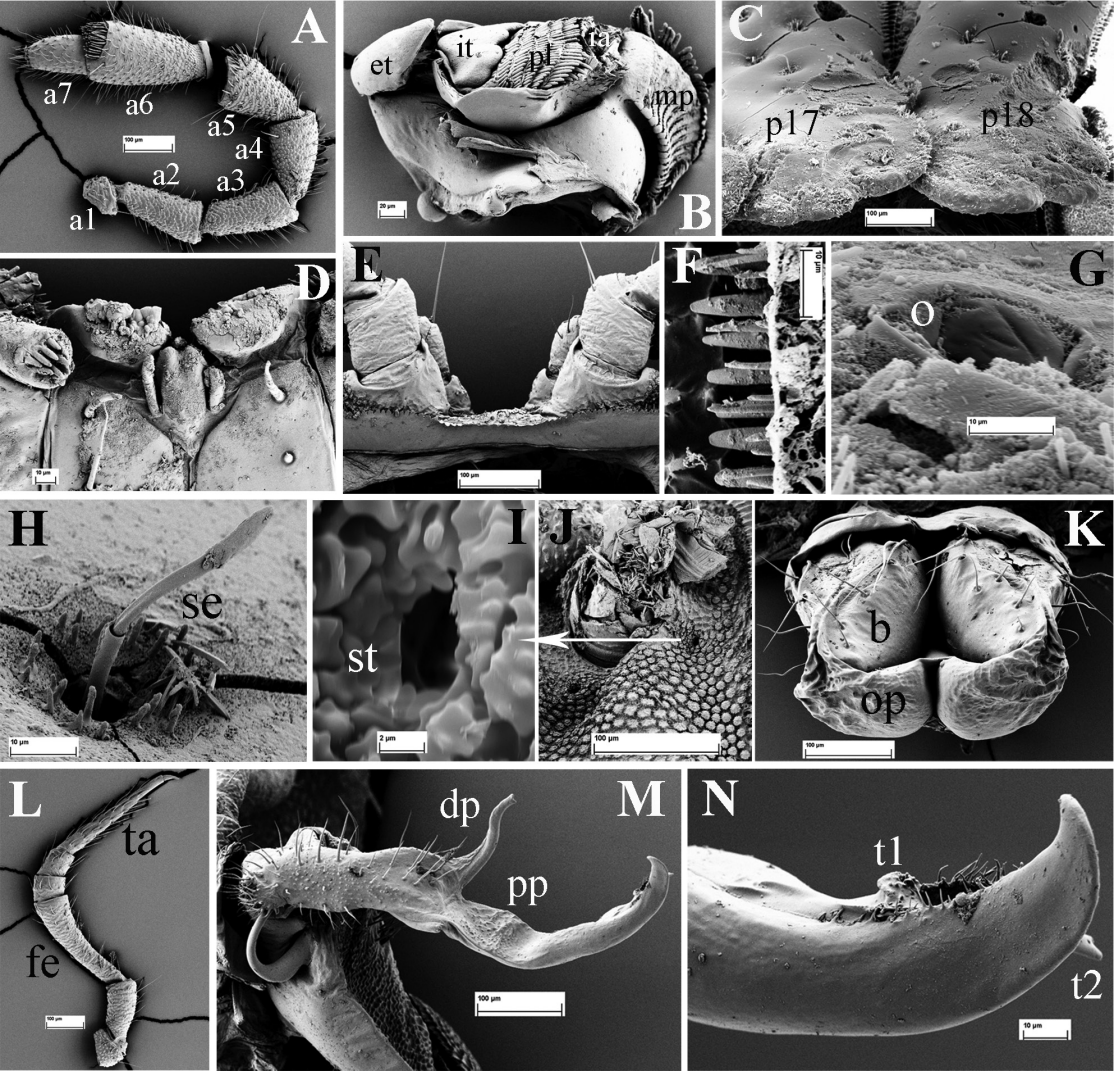
*Eutrichodesmus deporatus* sp. n., SEM, male paratype from Cave Tham Pathok. **A** right antenna, lateral view (a1–a7 = antennomeres 1–7) **B** right mandible, general view (et = external tooth; it = internal tooth; pl = pectinate lamellae; ia = intermediate area; mp = molar plate) **C** paratergum 17 (p17) and 18 (p18), dorsal view **D** tip of gnathochilarium, ventral view **E** sternum 6, caudal view **F** limbus of metatergum 14, dorsal view **G** ozopore (o) on paratergum 17 **H** a seta (se) on metatergum 14, general view **I** stigmata (st), detail **J** stigmata of segment 17, subventral view **K** female paratype, vulvae, general view (op = operculum; b = bursa) **L** midbody leg, frontal view (fem = femur; ta = tarsus) **M** right gonopod, mesal view (dp = distofemoral process; pp = papillate process) **N** tip of right gonopod, mesal view (t1–2 = teeth 1–2).

Head (Fig. 5A), bacilliform sensilla on antennae (Fig. 6A), gnathochilarium (Fig. 6D), mandibles (Fig. 6B), prozona (Fig. 5E), endoterga, metatergal setae (Fig. 6H), sterna (Fig. 6E), pleurosternal keels, stigmata (Fig. 6I–J), legs (Fig. 6K), gonopod aperture, telson (Fig. 5I–J), and vulvae (Fig. 6K) all similar to *Eutrichodesmus steineri* sp. n.

Collum subtrapeziform, with five transverse rows of round microvillose tubercles, flattened medially (Fig. 5D). Fore margin with two distinct tubercles on each side (Fig. 5D).

Stricture between pro- and metazona broad and shallow, finely microgranulate (Fig. 5E). Limbus with relatively long crenulations and nearby abundant microvilli (Fig. 6F).

Metaterga 2–5 with three transverse mixostictic rows of similarly microvillose tubercles, flattened medially, about 7 + 7 per row (Fig. 5B–D). Three transverse rows of rather small, flat tuberculations on metaterga 6–19 (Fig. 5F–H). Metaterga 5–19 each with a very high, large, bifid, mid-dorsal projection (Fig. 5B, F–H). Projections 5–17 upright, then directed slightly caudad on matetaga 18 and 19 (Fig. 5F).

Front margin of paraterga 2–4 strongly elevated (Fig. 5B–D). Paraterga 2 strongly enlarged, vaguely trilobate laterally, with four frontal and three caudal evident lobulations (Fig. 5D); paraterga 3 and 4 slightly shorter than others, bilobate laterally (Fig. 5B–C); following paraterga laterally 3- or 4-lobulated, caudolaterally at base with two distinct lobulations (Figs 5F, H, 6C).

Ozopores mostly reduced, only visible on paraterga 17 (Fig. 6G).

**Figure 7.**
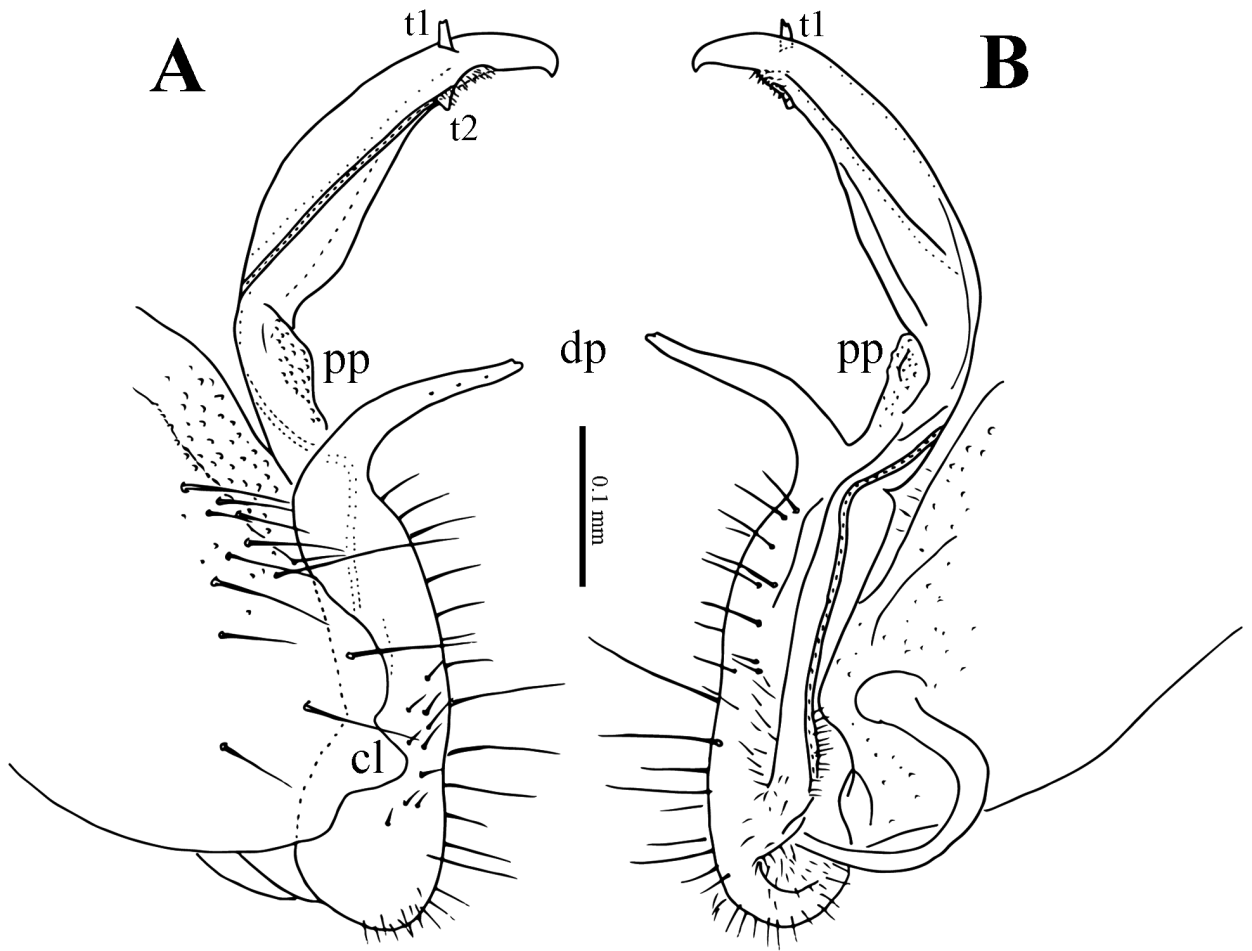
*Eutrichodesmus deporatus* sp. n., ♂ paratype from Cave Tham Pathok. **A–B** right gonopod, lateral and mesal views, respectively. Abbreviations: cl = coxal lobe; dp = distofemoral process; pp = papillae process; t1–2 = teeth 1–2.

Gonopods (Figs 6M–N, 7) simple. Coxae abundantly micropapillate and sparsely setose ventrolaterally, with an apicolateral lobe (**cl**). Telopodite longer than coxite, slender throughout, setose in basal half, with a prominent, digitiform, lateral, distofemoral process (**dp**) at about midway. Acropodite with a micropapillate process (**pp**) at base and a dorsolateral tooth (**t1**) subapically, tip slightly bifid; seminal groove terminating in a hairpad at a small triangular tooth (**t2**) subapically.

##### Remarks.

The specimens come from the Cave Tham Pathok which is about 100 meters long, and the temperature inside is about 15 °C. The animals were found living at a small waterfall at a distance of 20 meters from the entrance. In addition, *Heteropoda* spp. (Arachnida) and *Glyphiulus* sp. (Diplopoda, Cambalopsidae) were found in the cave (Steinmetz 2007).

The pallid body and long legs suggest that *Eutrichodesmus deporatus* sp. n. is most likely a troglobite.

#### Eutrichodesmus paraster

Taxon classificationAnimaliaPolydesmidaHaplodesmidae

Liu & Wesener sp. n.

http://zoobank.org/7EC7CE3B-1990-49E5-953C-B0B8D5B46DEB

**Figure 8.**
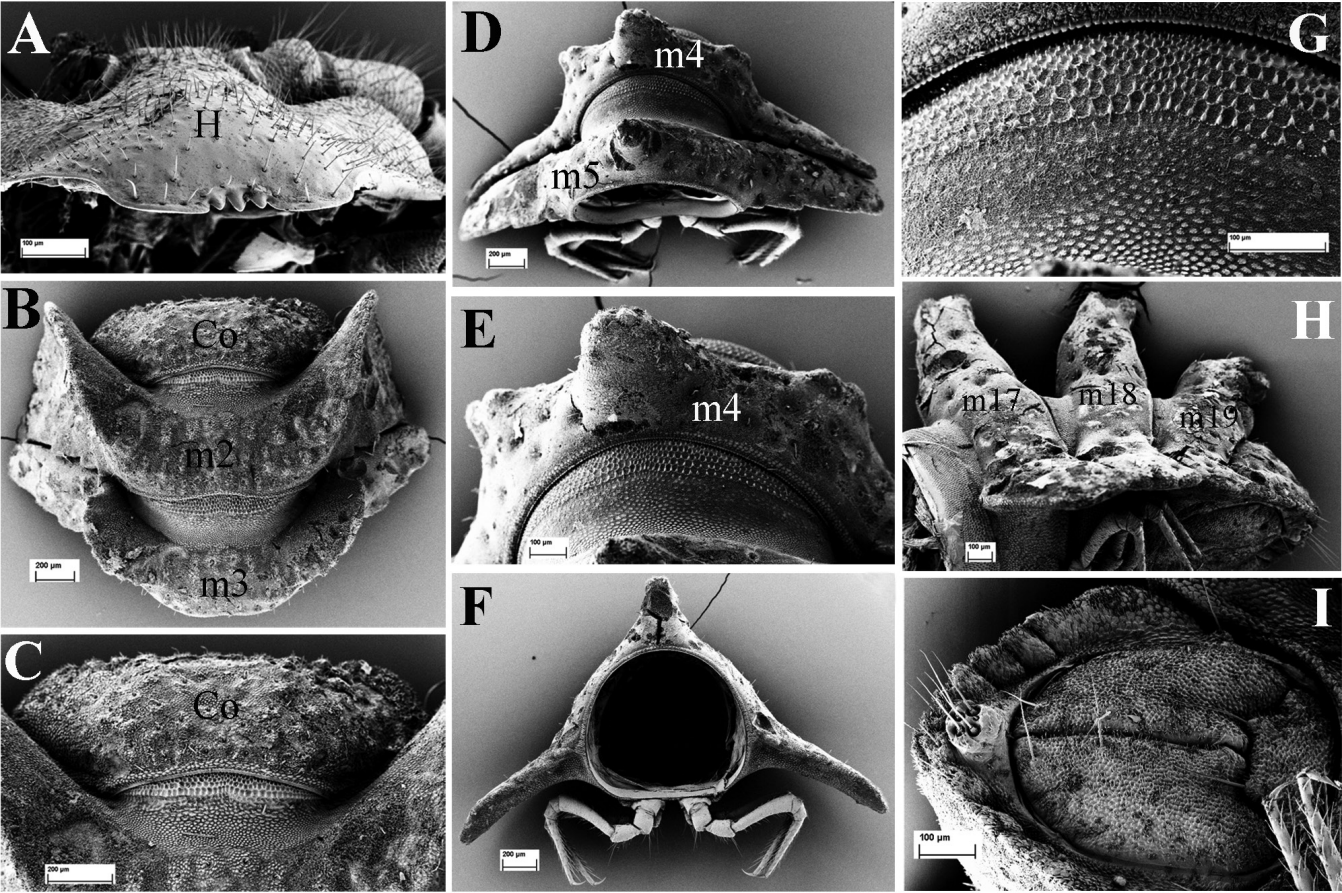
*Eutrichodesmus paraster* sp. n., SEM, male holotype from Cave Tham Long Puang. **A** head (H), subfrontal view **B** collum (Co) and segments 2–3 (m2–m3 = metaterga 2, 3), dorsal view **C** collum (Co), dorsal view **D** segments 4 and 5 (m4–m5 = metaterga 4, 5), dorsal view **E** segment 4 (m4 = metatergum 4), dorsal view **F** cross-section of segment 6, caudal view **G** prozonum 5, dorsal view **H** segments 17–19 and telson, lateral view (m17–m19 = metaterga 17–19) **I** telson, ventral view.

**Figure 9.**
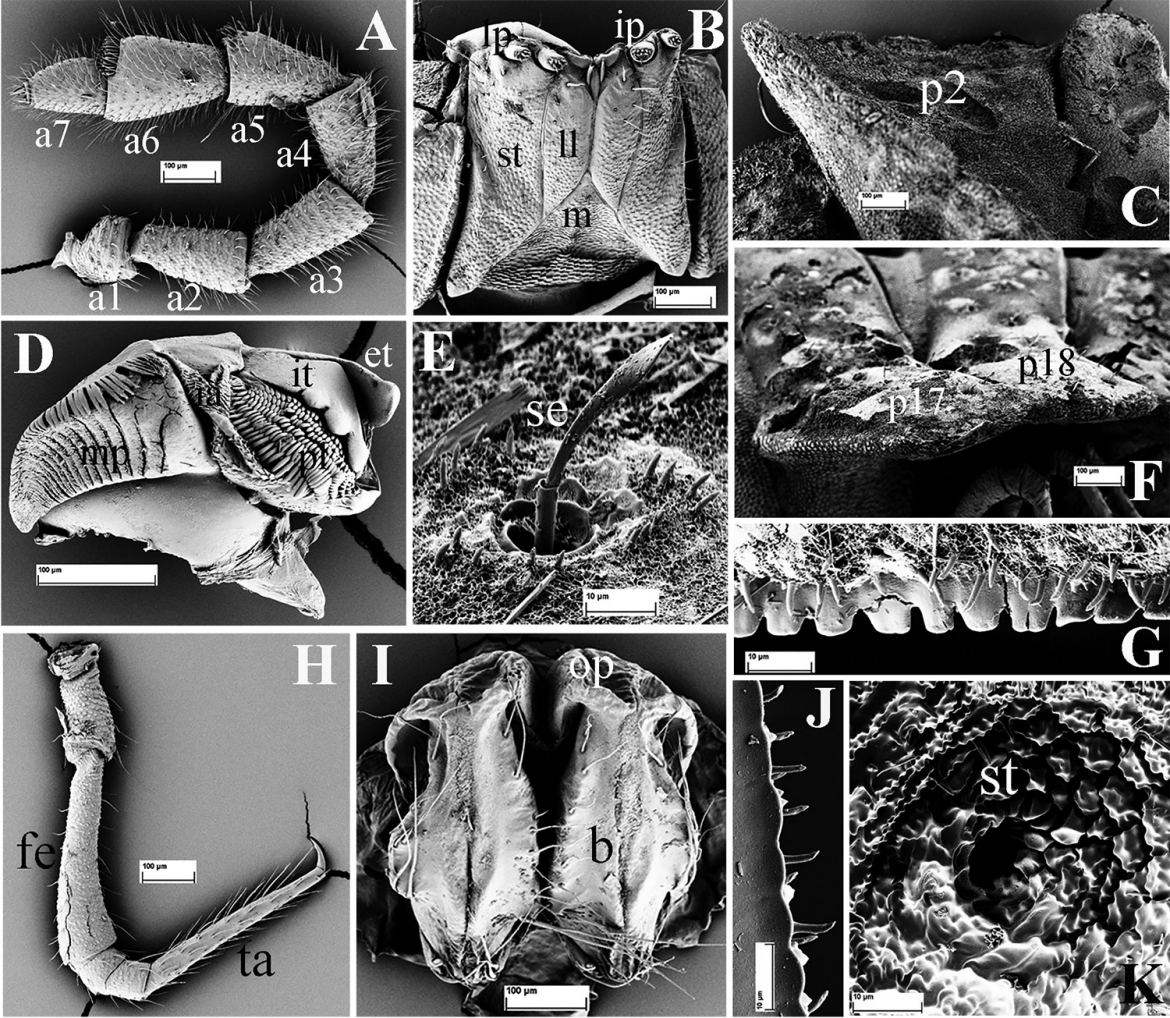
*Eutrichodesmus paraster* sp. n., SEM, male holotype from Cave Tham Long Puang. **A** right antenna, lateral view (a1–a7 = antennomeres 2–7) **B** gnathochilarium, ventral view (lp = lateral palpus; ip = inner palpus; st = stipites; ll = lamellae linguales; m = mentum) **C** paratergum 2 (p2), dorsal view **D** right mandible, general view (et = external tooth; it = internal tooth; pl = pectinate lamellae; ia = intermediate area; mp = molar plate) **E** a seta (se) on metatergum 18 **F** paraterga 17 (p17) and 18 (p18), lateral view **G** limbus of metatergum 5, dorsal view **H** midbody leg, frontal view (fe = femur; ta = tarsus) **I** female paratype, vulvae, general view (op = operculum; b = bursa) **J** endotergum 5 **K** stigmata (st), ventral view.

**Figure 10.**
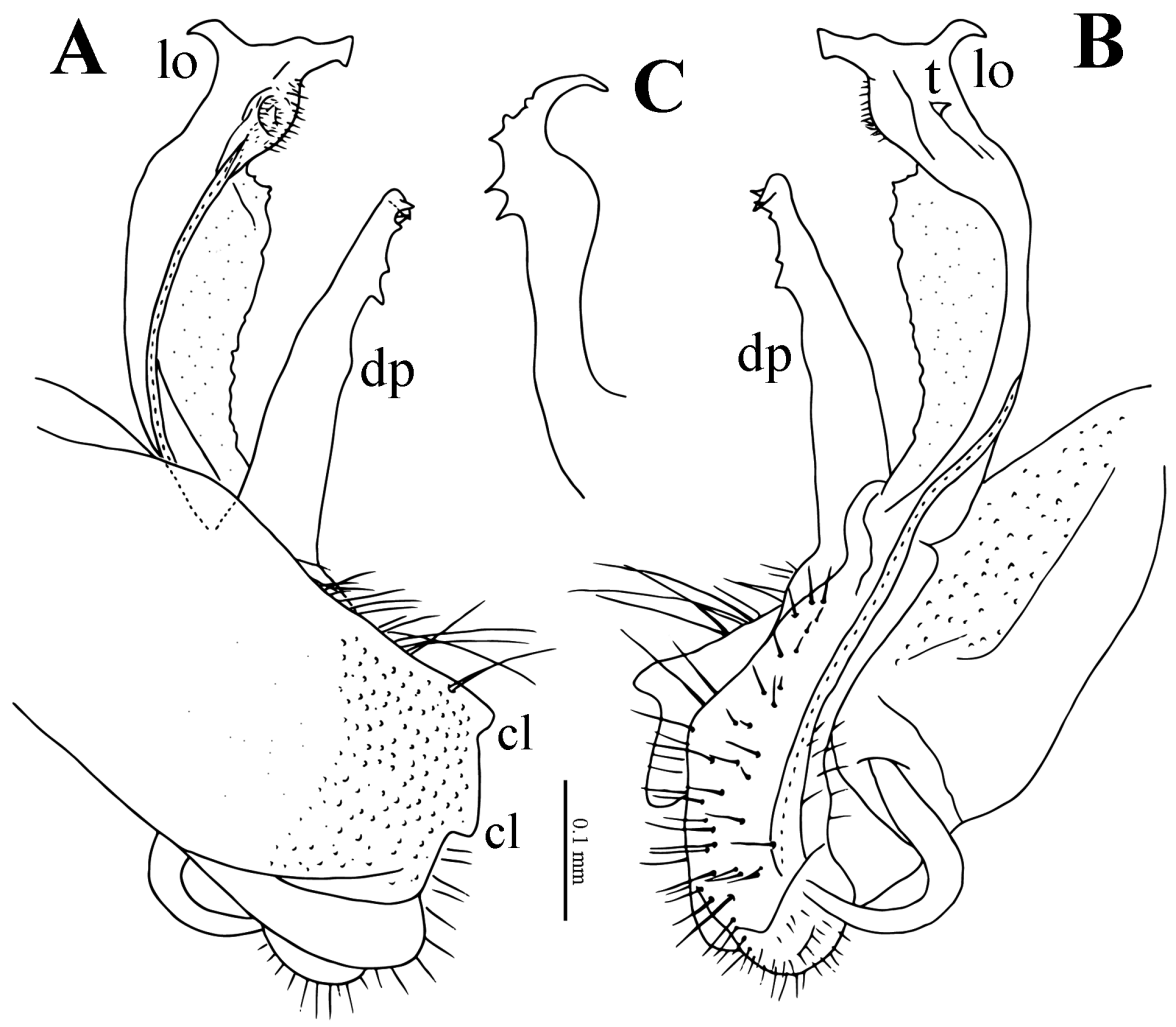
*Eutrichodesmus paraster* sp. n., male holotype from Cave Tham Long Puang. **A–B** right gonopod, lateral and mesal views, respectively **C** distofemoral process, subventral view. Abbreviations: cl = coxal lobe; dp = distofemoral process; lo = lobule; t = tooth.

Figs 1C , 8 , 9 , 10

##### Material examined.

Holotype male (SEM), (SMF), Laos, Huaphan Prov., Xop, Cave Tham Long Puang (F 48-123-001), N20°28'25.7", E103°21'44.4", 16.I.2009, coll. H. Steiner (101/09-).

##### Paratypes.

1 female (SMF), same data as holotype; 1 juvenile (ZFMK MYR6131), same data.

##### Etymology.

To emphasize the similarity to *Eutrichodesmus aster* Golovatch, Geoffroy, Mauriès & VandenSpiegel, 2009; adjective.

##### Diagnosis.

Differs from other species of the genus primarily by the completely reduced ozopores. Superficially very similar to *Eutrichodesmus aster*, but distinguished from the latter through the smaller body, laterally 3-lobulated paraterga, and the relatively complex gonopod showing a large, laterally denticulate, distofemoral process; the acropodite subapically has a very small mesal tooth and an evident, digitiform, dorsal lobule. See also Key above.

##### Description.

Length of adults ca. 8.0 mm (holotype) or 9.0 mm (paratype), width 1.0 mm and 2.5 mm on midbody pro- and metazona, respectively.

Coloration uniformly pallid (Fig. 1C).

Adults with 20 segments (Fig. 1C), body conglobation complete.

Antennae short, but slender; in length, antennomere 6 = 3 > 2 > 4 = 5 > 7 > 1 (Fig. 9A).

Labrum with three teeth (Fig. 8A).

Head (Fig. 8A), bacilliform sensilla on antennae (Fig. 9A), gnathochilarium (Fig. 9B), mandibles (Fig. 9D), prozona (Fig. 8G), endoterga (Fig. 9J), metatergal setae (Fig. 9E), sterna (Fig. 8F), pleurosternal keels, stigmata (Fig. 9K), legs (Fig. 9H), gonopod aperture, telson (Fig. 8I) and vulvae (Fig. 9I) all similar to those in *Eutrichodesmus steineri* sp. n.

Collum subtrapeziform, with six transverse rows of round microvillose tubercles (Fig. 8B–C).

Stricture between pro- and metazona broad and shallow, more finely alveolate-microgranulate than prozona (Fig. 8G). Limbus regularly microcrenulate (Fig. 9G).

Front margin of metaterga 2–4 strongly elevated, each latter with three transverse mixostictic rows of similar tubercles (Fig. 8B, D–E). Following metaterga with three transverse rows of small, flattened, microvillose tuberculations (Figs 8D, 9E). Metaterga 4–19 each with a very high mid-dorsal projection, slightly smaller on metatergum 4 (Fig. 8D–F, H); tip of projections usually bilobed, always bilobed on each side on metaterga 5 and 6 (Fig. 8D). Projections upright, directed slightly caudad only on metatergum 19 (Fig. 8H).

Paraterga 2 strongly enlarged, vaguely 4-lobulated laterally (Fig. 9C). Following paraterga bi- or trilobate laterally in anterior and posterior parts of body, respectively, each with two small caudal lobulations (Figs 1C, 9F).

Ozopores absent.

Gonopods (Fig. 10) relatively complex. Coxae large, micropapillate and sparsely setose ventrolaterally, with two small apicolateral lobes (**cl**). Telopodite slightly longer than coxite, slender throughout, setose in basal half, with a prominent, laterally denticulate, distofemoral process (**dp**) at about midway. Acropodite twisted, subapically with a very small mesal tooth (**t**) and an evident digitiform lobule (**lo**) dorsally; seminal groove terminating subapically in a hairpad.

##### Remarks.

As this species was collected in a cave, and has a pallid body it appears to be a real troglobite. The absence of ozopores is unique for the family Haplodesmidae.

#### Eutrichodesmus parvus

Taxon classificationAnimaliaPolydesmidaHaplodesmidae

Liu & Wesener sp. n.

http://zoobank.org/09C6E621-12C0-4837-A231-99A692E135C2

**Figure 11.**
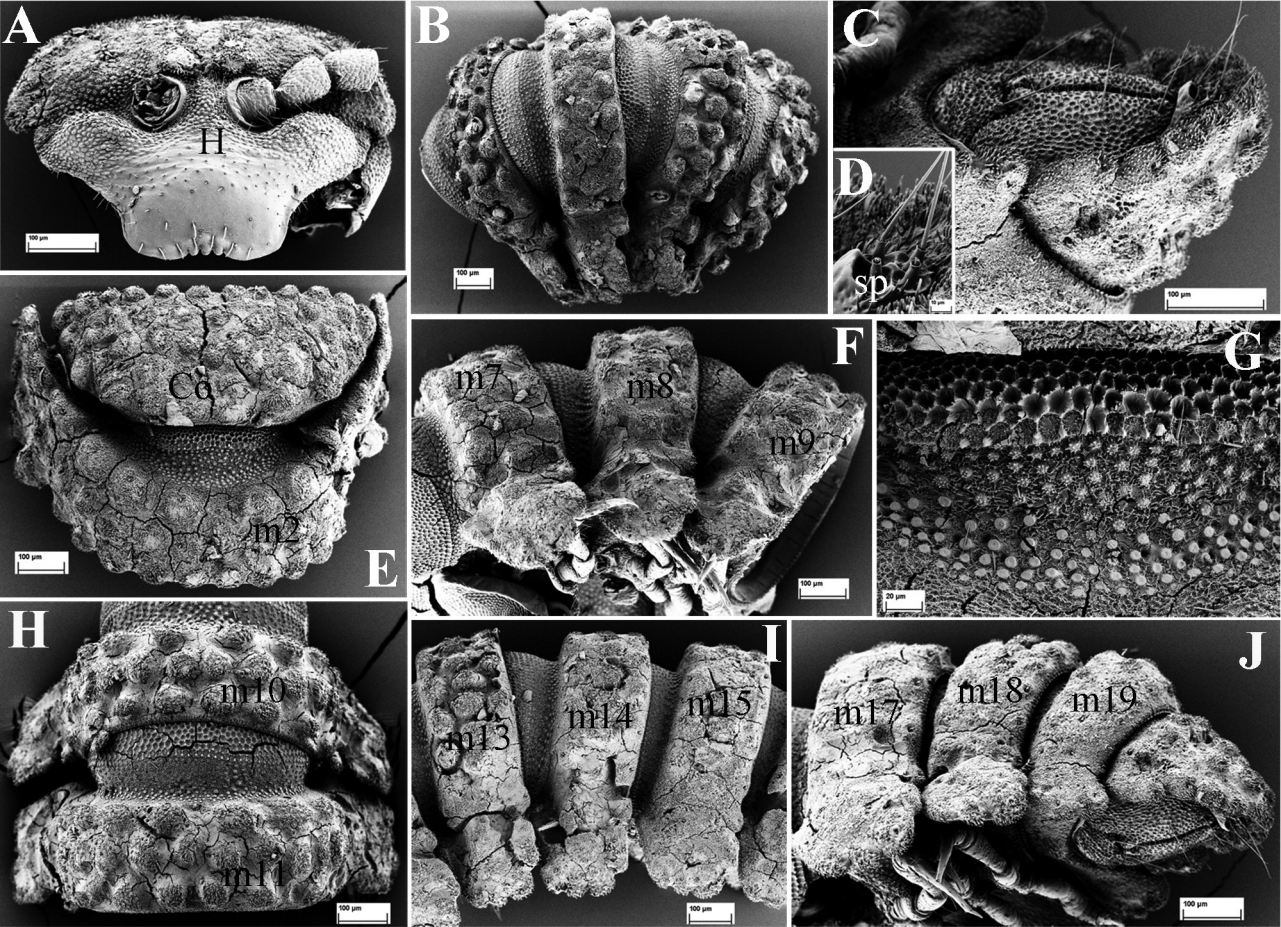
*Eutrichodesmus parvus* sp. n., SEM, male paratype from Cave Tham Nam Long. **A** head (H), frontal view **B** segments 3–6, subdorsal view **C** telson, subventral view **D** spinneret (sp), detail **E** collum (Co) and segment 2 (m2 = metatergum 2), dorsal view **F** segments 7–9, lateral view (m7–m9 = metaterga 7–9) **G** prozonum 2, dorsal view **H** segments 10–11, dorsal view **I** segments 13–15, dorsal view **J** segments 17–19 and telson, lateral view (m10–11, 13–15, 17–19 = metaterga 10, 11, 13–15, 17–19).

**Figure 12.**
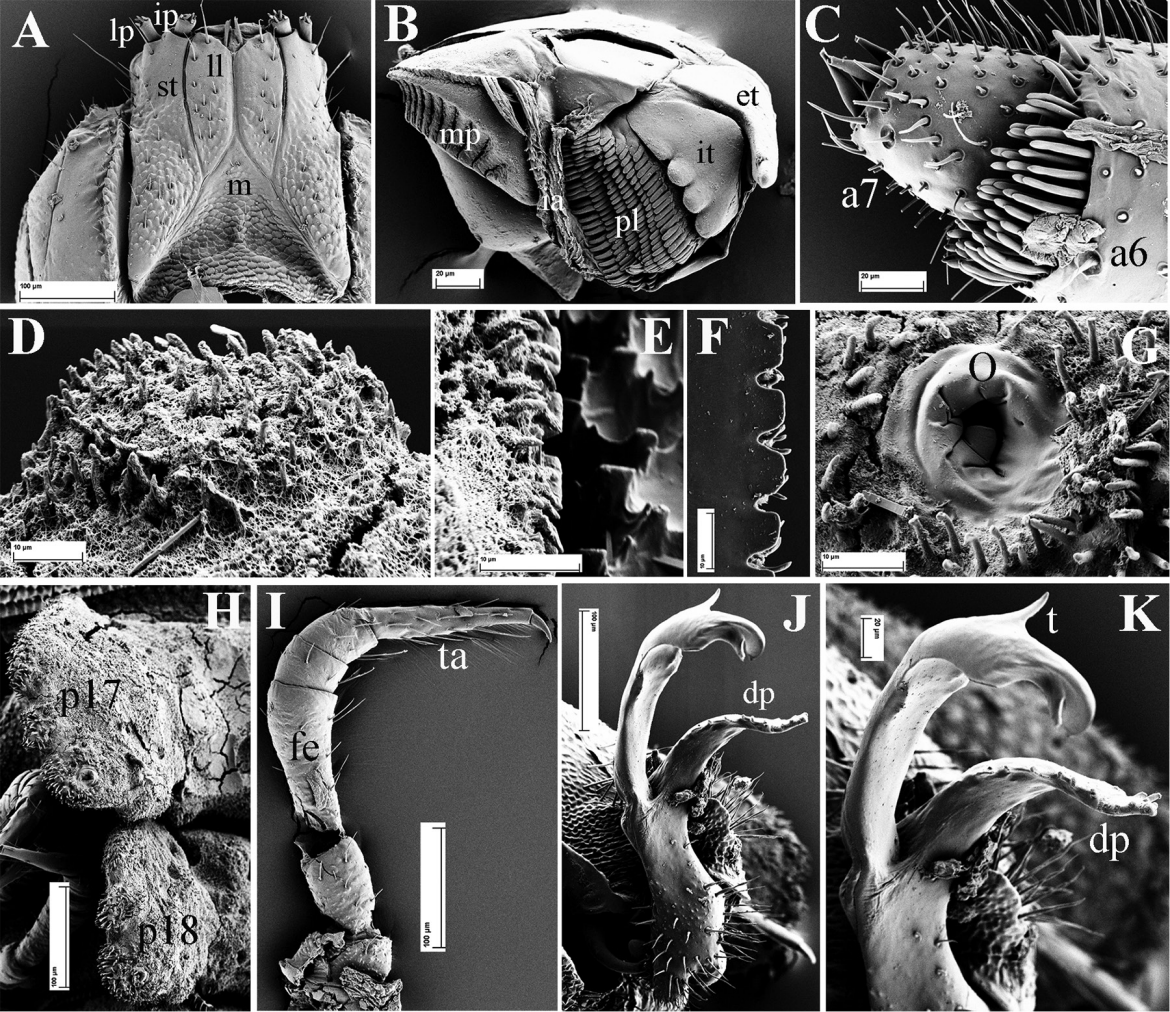
*Eutrichodesmus parvus* sp. n., SEM, male paratype from Cave Tham Nam Long. **A** gnathochilarium, ventral view (lp = lateral palpus; ip = inner palpus; st = stipites; ll = lamellae linguales; m = mentum) **B** right mandible, general view (et = external tooth; it = internal tooth; pl = pectinate lamellae; ia = intermediate area; mp = molar plate) **C** tip of antenna, laterodorsal view (a6–a7 = antennomeres 6, 7) **D** a tubercle at fore margin of collum, dorsal view **E** limbus of metatergum 10, dorsal view **F** endotergum 9 **G** ozopore (o) on paratergum 17, general view **H** paratergum 17 (p17) and 18 (p18), lateral view **I** midbody leg, frontal view (fe = femur; ta = tarsus; cl = claw) **J** left gonopod, submesal view **K** half of left gonopod, detail (dp = distofemoral process; t = tooth).

**Figure 13.**
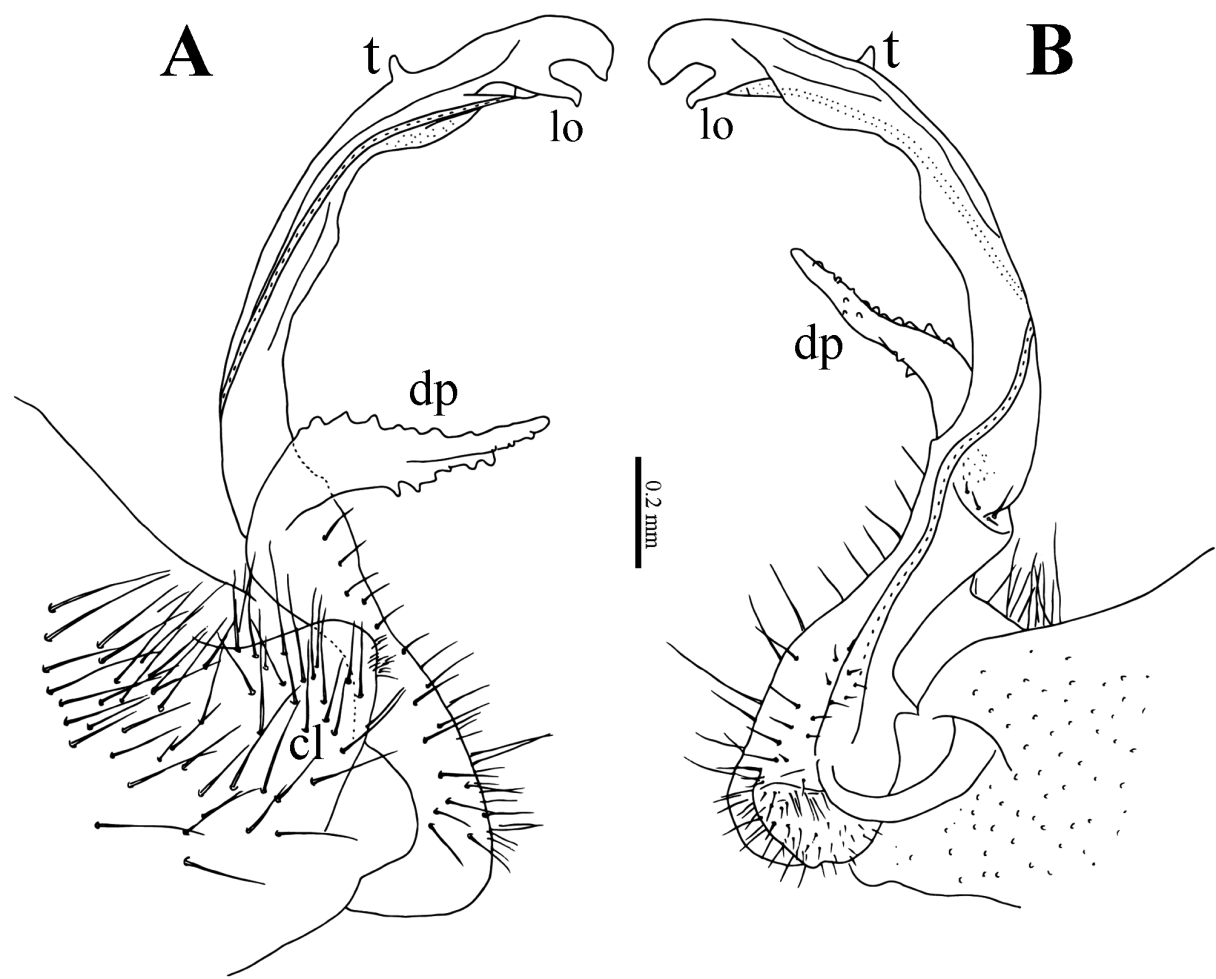
*Eutrichodesmus parvus* sp. n., male paratype from Cave Tham Nam Long. **A–B** right gonopod, lateral and mesal views. Abbreviations: cl = coxal lobe; dp = distofemoral process; lo = lobe; t = tooth.

Figs 1D , 11 , 12 , 13

##### Material examined.

Holotype male (SMF), Laos, Huaphan Prov., Cave Tham Nam Long (F 48-125-007), N 20°27'50.3", E 104°9'10.7", 10.I.2008, coll. H. Steiner (133/08-).

##### Paratypes.

1 male (SEM), (ZFMK MYR6132), 1 female (ZFMK MYR6128), same data as holotype; 2 females (SMF), same data.

##### Etymology.

To emphasize the very small body of this species; adjective.

##### Diagnosis.

Differs from other species of the genus in the very small body (4.0–5.0 mm long), three regular transverse rows of round microvillose tubercles on metaterga, short paraterga, as well as the relatively complex gonopod with a large, laterally denticulate, distofemoral process; the acropodite subapically has a small dorsal tooth and an evident, digitiform, ventral lobe; the seminal groove is devoid of a hairpad near the place of its termination. See also Key above.

##### Description.

Length of adults of both sexes ca .4.0–5.0 mm, width 0.3–0.4 mm and 0.6–0.8 mm on midbody pro- and metazona, respectively.

Coloration uniformly light yellow-brown with pallid antennae (Fig. 1D).

Adults with 20 segments (Fig. 1D), body subcylindrical, conglobation incomplete.

Antennae short (Fig. 1D); in length, antennomere 6 > 5 > 2 > 3 = 4 > 7 > 1.

Labrum with three teeth (Fig. 11A).

Head (Fig. 11A), bacilliform sensilla of antenna (Fig. 12C), gnathochilarium (Fig. 12A), mandible (Fig. 12B), prozona (Fig. 11G), endoterga (Fig. 12F), sterna, pleurosternal keels, gonopod aperture (Fig. 11F), telson (Fig. 11C–D) and vulvae all similar to those in *Eutrichodesmus steineri* sp. n.

Collum semi-circular, with five transverse rows of round, small, microvillose tubercles (Fig. 11E). First row with 12 round tubercles (Fig. 11E).

Stricture between pro- and metazona broad and shallow, more finely alveolate-microgranular than prozona (Fig. 11G). Limbus regularly microcrenulate (Fig. 12E).

Metaterga 2 to pre-anal segment each with three transverse rows of high, round, regular, microvillose tubercles, usually about 5 + 5 per row (Figs 11–12). Metatergal setae inconspicuous, mostly abraded.

Paraterga short, slightly extending down below level of venter, especially paraterga 18 and 19 being shorter with previous one (Figs 11J, 12H); usually trilobate laterally and with two caudal lobulations (Figs 11F, I, J, 12H).

Pore formula normal, ozopores distinct, located near top of caudolateral lobulation (Fig. 12G–H).

Legs long and slender, femur somewhat longer than tarsus (Fig. 12I).

Gonopods (Figs 12J–K, 13) relatively complex. Coxae large, micropapillate and setose ventrolaterally, with a large apicolateral lobe (**cl**). Telopodite slightly longer than coxite, slender throughout, setose in basal half, with a large, prominent, denticulate, lateral, distofemoral process (**dp**) at about midway. Acropodite subapically with a small tooth (**t**) dorsally and an evident digitiform lobe (**lo**) ventrally; seminal groove terminating without hairpad.

#### Eutrichodesmus

Taxon classificationAnimaliaPolydesmidaHaplodesmidae

sp.

##### Material examined.

1 female, 2 juveniles (SMF), Laos, Cave Tham Mokfek, N20°48'34.7", E101°47'14.5", 28.I.2010, coll. H. Steiner (155/10-).

##### Remarks.

These specimens do not fit any of the six *Eutrichodesmus* described from Laos, base on somatic characters alone. As only one adult female is available, we refrain from naming this species.

## Discussion

All attempts to extract the DNA from some of the specimens using the DNeasy Blood & Tissue kit from Qiagen were unsuccessful. One reason might be the specimens had been preserved in low-concentration ethanol for nearly ten years. In addition, it was not at all easy to get enough tissue from legs of these tiny specimens for DNA extractions.

In our study, two new species were found, *Eutrichodesmus deporatus* sp. n. and *Eutrichodesmus paraster* sp. n., both from caves, and unsurprisingly less pigmented, that show strongly or completely reduced ozopores. The function of the defensive glands and their ozopores is known to lie in the production of defence fluids, as a protection against predators (Shear 2015). From the recent paper concerning the adaptation in the cave millipedes to the cave environment (Liu et al. 2017) it remains unclear whether or not the suppression may be related to cavernicoly. We are rather inclined to think it is not. Firstly, about half of the known species of *Eutrichodesmus* are cave-dwellers, but their pore formulae are normal: 5, 7, 9, 10, 12, 13, 15–19 (Golovatch et al. 2009a, 2009b, 2015, 2016a; Liu and Tian 2013). Secondly, within the family Haplodesmidae the normal pore formulae dominate, but there are several genera or species with increased formulae as well: *Helodesmus* Cook, 1896 and *Koponenius* Golovatch & VandenSpiegel, 2014, both showing 5, 7–17(18) formulae, and culminating in *Prosopodesmus panporus* Blower & Rundle, 1980 with its unique 5–17(18) formula (Golovatch et al. 2009a, Mesibov 2012; Golovatch and VandenSpiegel 2014). The occasional loss of ozopores in haplodesmids seems to be surprising, but not unthinkable. After all, some species of Sphaeriodesmidae have also been noted to lack ozopores. In addition, ozopores are often very difficult to observe in those *Eutrichodesmus* species which lack porosteles and where the small ozopores open flush with a surface beset with tubercles, grains, setiferous fossae and microvilli.

All material of *Eutrichodesmus* from Laos treated here was collected opportunistically by a research group focusing on Arachnida, headed by Peter Jäger (SMF). Because *Eutrichodesmus* species hide in the soil and are small and often coiled, it is possible they were missed during searches focused on Arachnida. It can only be hoped that more efforts to investigate and describe the highly unique and diverse diplopod fauna of Laos will be undertaken in the future, before the utilization of natural resources leads to the loss of the existing natural forests, something that has already happened in the last decades in neighbouring nations (Sodhi et al. 2010).

## Supplementary Material

XML Treatment for Eutrichodesmus steineri

XML Treatment for Eutrichodesmus deporatus

XML Treatment for Eutrichodesmus paraster

XML Treatment for Eutrichodesmus parvus

XML Treatment for Eutrichodesmus
